# Genome-wide burden of deleterious coding variants increased in schizophrenia

**DOI:** 10.1038/ncomms8501

**Published:** 2015-07-09

**Authors:** Loes M. Olde Loohuis, Jacob A. S. Vorstman, Anil P. Ori, Kim A. Staats, Tina Wang, Alexander L. Richards, Ganna Leonenko, James T. Walters, Joseph DeYoung, GROUP consortium, René S. Kahn, Don Linszen, Jim van Os, Durk Wiersma, Richard Bruggeman, Wiepke Cahn, Lieuwe de Haan, Lydia Krabbendam, Inez Myin-Germeys, Rita M. Cantor, Roel A. Ophoff

**Affiliations:** 1 Center for Neurobehavioral Genetics, University of California Los Angeles, Los Angeles, California 90095, USA; 2 Department of Psychiatry, Brain Center Rudolf Magnus, University Medical Center Utrecht, Utrecht 3584 CG, The Netherlands; 3 MRC Centre for Psychiatric Genetics and Genomics, Cardiff University, Cardiff CF24 4HQ, UK; 4 Department of Human Genetics, University of California Los Angeles, Los Angeles 90095, USA; 5 Department of Psychiatry, Rudolf Magnus Institute of Neuroscience, University Medical Center Utrecht, 3584 CG Utrecht, The Netherlands.; 6 Department of Psychiatry, Academic Medical Center, University of Amsterdam, 1105 AZ Amsterdam, The Netherlands.; 7 Mental Health Research and Teaching Network, Maastricht University Medical Center, South Limburg, 6200 MD Maastricht, The Netherlands.; 8 Department of Psychiatry, University Medical Center Groningen, University of Groningen, 9713 GZ Groningen, The Netherlands.; 9 Department of Educational Neuropsychology, VU University Amsterdam, 1081 BT Amsterdam, The Netherlands.

## Abstract

Schizophrenia is a common complex disorder with polygenic inheritance. Here we show that by using an approach that compares the individual loads of rare variants in 1,042 schizophrenia cases and 961 controls, schizophrenia cases carry an increased burden of deleterious mutations. At a genome-wide level, our results implicate non-synonymous, splice site as well as stop-altering single-nucleotide variations occurring at minor allele frequency of ≥0.01% in the population. In an independent replication sample of 5,585 schizophrenia cases and 8,103 controls of European ancestry we confirm an enrichment in cases of the alleles identified in our study. In addition, the genes implicated by the increased burden of rare coding variants highlight the involvement of neurodevelopment in the aetiology of schizophrenia.

## Abstract (web summary)

Schizophrenia is a complex disorder with high heritability but poorly understood genetics. Here Olde Loohuis *et al.* compare schizophrenia patients to unaffected individuals and identify an increased individual burden of rare deleterious mutations in patients.

## Graphical abstract

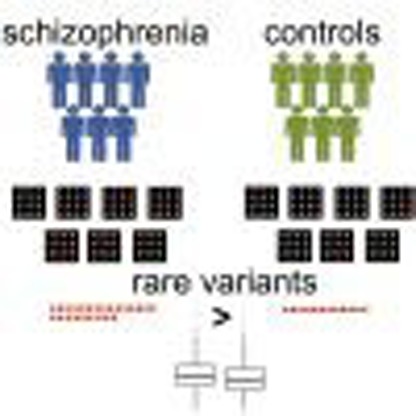

Despite the high heritability of schizophrenia, identifying the contributing genetic variants has been a daunting challenge. In addition to common variants that continue to be revealed by conventional genome-wide association studies (GWAS)^1^, rare variants, including *de novo* mutations^2^,^3^ and genomic copy-number variants^4^,^5^, have been found to play a role in disease risk. However, due to their low minor allele frequencies (MAFs) and modest effect sizes, it is difficult to establish associations with rare SNVs using standard association tests. For this reason, we investigated the burden of rare SNVs in schizophrenia by an individual set-unique burden (ISUB) analysis of coding variants that occur in cases or controls, but not both. While our method does not rely on frequency thresholds, in practice, ISUB includes variants with MAFs ranging from 0.05 to 0.6% in our study, and with a mean of 0.05% in the population. A previous finding suggests enrichment of primarily singleton nonsense variants in schizophrenia in a set of pre-selected genes^6^. In contrast, we examine a genome-wide distribution not limited to a subset of genes previously associated with the disease. In addition, instead of studying extremely rare loss-of-function variants we include rare but recurrent variants in the population (MAF>1:10,000) with milder deleteriousness criteria. Because of this approach, we are able to study the individual cumulative burden rather than burden across groups of cases and controls. To quantify the individual burden of variants in our sample, we incorporated scores from a previously developed CONsensus DELeteriousness (CONDEL) algorithm^7^ aimed at assessing the impact of non-synonymous SNVs on protein function.

Our results show an increased burden of rare deleterious coding variants in cases versus controls. Our findings implicate non-synonymous as well as stop-altering and splice site SNVs at a genome-wide level. Functional enrichment analysis of genes impacted by rare variant burden highlights the involvement of neurodevelopment in the aetiology of schizophrenia. Finally, we observe no clear link between common and rare susceptibility alleles and their relative contribution to the disease. Our results underscore the polygenic nature of schizophrenia across the allelic spectrum.

## Results

### Sample description and variant selection

**Figure 1:**
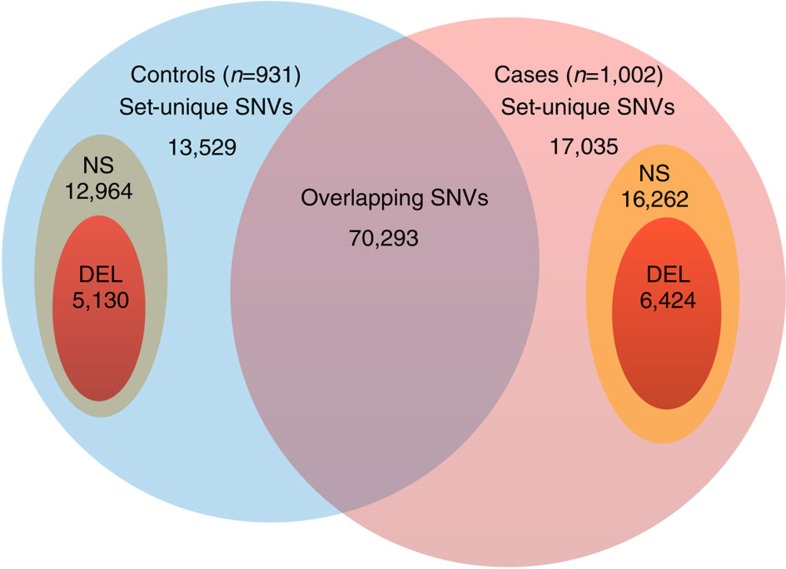
Set-unique SNVs. Diagram depicting the set-unique variants (not to scale, cases *n*=1,002, controls *n*=931). The set NS represents all variants that are scored by ISUB, and the set DEL includes only those variants that are predicted to be deleterious. In particular, the set DEL includes stop-altering and splice site variants.

We collected coding sequence variation in 1,042 schizophrenia patients and 961 controls using the Illumina HumanExome BeadChip array. From a total of 100,857 variants observed in our sample, 75,837 are protein-coding SNVs that were scored by ISUB. From these, 16,262 occurred only in cases, of which 6,424 are predicted to be deleterious. For controls, 12,964 were set-unique non-synonymous, with a total of 5,130 predicted to be deleterious (see Fig. 1). The number of observations ranges from 1 to 12 per SNV with a mean of 1.43 (median=1) in cases and a mean of 1.39 (median=1) in controls. The mean MAF of the set-unique variants included in our study is 0.05% in the population based on individuals of European Ancestry included in ESP6500.

### Increased burden of rare variants in schizophrenia

The genome-wide ISUB for deleterious variants is increased significantly in cases versus controls (empirical *P*=0.018) (see Table 1, Supplementary Fig. 1 and Supplementary Table 1). Although a similar difference is observed for the set of all non-synonymous variants (NS, empirical *P*=0.033), the signal is driven primarily by deleterious variants (DEL, empirical *P*=0.018) and not by the complementary set of non-synonymous variants predicted to be non-deleterious (NS—DEL, empirical *P*=0.492), as indicated by the information in Table 1 (Methods). Given the differences in rare variants between cases and controls, we investigate the nature, robustness and polygenicity of this observed increase. To illustrate, the increased burden may be caused by a larger number of variants per individual or by an increased predicted deleteriousness per SNV, or both. To this end, we characterized the quantitative/qualitative nature of the observed increase. Although not completely independent, our results suggest that the increased individual burden is primarily due to a larger number of rare deleterious SNVs (empirical *P*=0.015), rather than the SNVs in patients being more deleterious (empirical *P*=0.079, Supplementary Table 1).

In an independent replication sample of 5,585 schizophrenia cases and 8,103 controls of European ancestry we observe an increased frequency of the deleterious variants identified through ISUB: cases have an average of 3.4 of the total of 6,424 deleterious variants observed only in cases, compared with 3.3 in controls (empirical *P*=0.035).

These results are unlikely to represent an artefact caused by population stratification or cryptic relatedness for several reasons: (1) we performed stringent quality control (Supplementary Methods, and Supplementary Fig. 2); (2) ISUB score is a significant predictor of case–control status, even after including the first ten MDS components into the model (Supplementary Methods); and (3), we observe the same increased frequency in two independent samples of European Ancestry.

### Polygenic nature of the increased burden

To assess the polygenicity of the observed increase in burden as well as identify the contributions of the different types of variants, we next analysed the number of genes affected by the different types of variants. The variants we tested are (a) all deleterious variants, (b) splice site variants, (c) stop-altering variants and (d) so-called ‘double hits’. The latter includes genes with two or more non-synonymous SNVs within one individual, where we require at least one to be predicted to be deleterious. As summarized in Table 2, a greater number of genes are affected by deleterious rare variants in cases versus controls (4,533 genes in cases versus 3,795 in controls, empirical *P*=0.008, Table 2). More striking is that in cases an increased number of genes is affected by splice site variants (380 genes in cases versus 276 in controls, empirical *P*=0.016) as well as stop-altering stop-altering variants (350 and 253 genes respectively empirical *P*=0.004). We observe a trend in the number of genes with two or more non-synonymous SNVs (179 genes in cases, 103 in controls, empirical *P*=0.047), but this difference does not survive correction for multiple testing. To further investigate the polygenicity of rare variant burden, we excluded the variants in a set of genes shown in previous work to be most prominently enriched for rare disruptive mutations in schizophrenia (*n*=1,796)^6^. In this analysis, the observed difference in deleterious ISUB remains significant (empirical *P*=0.006) (Supplementary Methods).

### Functional classification of rare deleterious variants

To classify the genes implicated by ISUB analysis at a functional level, we examined tissue expression enrichment and pathway analysis using DAVID (database for annotation, visualization and integrated discovery)^8^. To restrict the total set of genes to a number suitable for DAVID analysis (*n*<3,000), we selected the genes with increased load of rare variants based on Fisher exact *P*-values (see Methods and Supplementary Methods for the precise definition of these genes with strongest evidence). This approach not only reduces the set of genes to a usable size (from 4,533 to 698 in cases), it also reduces noise from highly polymorphic genes with large number of non-synonymous variants seen in both cases and controls (for example, *NEB* in which 12 controls and 9 cases have at least one deleterious SNV, and *MUC16* with 6 controls and 12 cases^9^). Interestingly, we observed significant enrichment for genes expressed in fetal brain (uncorrected *P*=0.001, Benjamini corrected *P*=0.033, hypergeometric overlap test). To control for potential confounders such as gene length, we performed the same analysis in controls, and while some enrichments were observed, fetal brain genes were not significantly enriched (Supplementary Table 2). Pathway analysis points to the extracellular matrix (ECM) receptor interaction as the only significantly enriched pathway in cases (*P*=5.87E−05, Benjamini corrected *P*=8.30E−03, hypergeometric overlap test), whereas no pathway enrichment was observed in controls. The above results were confirmed by a permutation analysis sampling an equal number of cases and controls (931 individuals for both groups, Supplementary Table 3, Supplementary Methods).

### Relationship between common and rare susceptibility alleles

Given the increased individual burden of rare coding variants in schizophrenia, we next examined the relationship between common and rare susceptibility alleles. We tested the overlap between genes located in schizophrenia associated intervals from the GWAS study of the Psychiatric Genomics Consortium^1^ (ED Supplementary Table 3) and the genes implicated in our study. No significant overlap was observed in cases (83 overlap the 4,533 genes with deleterious alleles in cases versus 62/3,795 in controls, empirical *P*=0.070, Supplementary Table 2, Supplementary Methods). We also did not observe a correlation between ISUB score and sex (Supplementary Methods).

Quantifying the contribution of these variants to disease susceptibility, by odds ratios and similar statistics, is not suitable for low frequency alleles. For this reason, we measured the relative effect size by reduction in Nagelkerke’s *R*^2^ from logistic regression, and compared it with the relative impact of common SNVs using polygenic risk scores of the most recent GWAS results^1^. These regressions showed relative effect sizes of 10.7% for GWAS common alleles compared with 0.6% for rare deleterious alleles identified through ISUB (Supplementary Methods). Thus, common alleles contribute an order-of-magnitude more to disease susceptibility than the rare deleterious variants, in line with evidence presented by Purcell, *et al.*^6^. When comparing the polygenic risk score of cases with ISUB scores directly, no correlation was observed (Supplementary Methods). This suggests that common and rare disease risk alleles may be independently and additively enriched in cases versus controls.

Finally, we tested the overlap between our gene sets and the set of genes containing previously reported *de novo* mutations in schizophrenia^2^,^3^,^10^,^11^. While the difference between cases and controls is not significant (empirical *P*=0.070), we observe a highly significant overlap within each group (*P*<2.2e−16 for cases and *P*=4.44e−16 for controls, Supplementary Methods, hypergeometric overlap test).

## Discussion

Our results demonstrate the contribution of non-synonymous rare variants to the aetiology of schizophrenia. In the same frequency spectrum of previously observed rare variants, we identify not only a significant genome-wide increased individual burden of deleterious non-synonymous variants, but also an increased burden of stop-altering and splice site variants.

The deleterious variants identified through our analysis also occur at an increased frequency in cases compared with controls in an independent replication sample. Thus, not only does the increased burden of rare variants contribute to schizophrenia, also the specific variants implied by our analysis play a role in the aetiology of schizophrenia. We recognize that this is not a full replication of the main result of this study, since no formal ISUB analysis was performed on the replication sample. However, the result provides strong evidence with regard to the contribution of rare coding variants to the disease.

Previous work by Purcell *et al.*^6^ demonstrates enrichment of primarily ultra-rare nonsense variants in schizophrenia in a set of pre-selected genes. Our results also implicate non-synonymous and splice site SNVs at a genome-wide level. In addition, our analysis targets variants from a relatively rare allele frequency spectrum, not including ultra-rare variants. As a result, most individuals carry multiple rare SNVs, enabling us to examine the individual cumulative burden.

We chose to study ISUB variants rather than including variants based on allele frequency thresholds to maximize detection of the true signal from the rare variants contributing to disease. Limiting the alleles to singletons, for example, only diminishes the signal by excluding variants occurring more than once in either cases or controls. Alternatively, imposing a frequency threshold may increase noise by including variants with an equal MAF in both groups. Examining variants unique to either cases or controls, but without imposing a frequency threshold, is an appealing intermediate for studying rare variant burden. ISUB analysis is especially advantageous when studying sample and variant sets of a limited size, such as our sample, as it generates a more informative signal than one obtained from setting extreme frequency thresholds. As sample sizes increase beyond a certain size, however, the power to assess individual burden by ISUB may decrease. Under those circumstances, different selection criteria of SNVs and genes are needed. We propose the approach of selecting variants below a certain frequency threshold based on association *P* values.

The observed enrichment of genes expressed in fetal brain fits well within the existing body of evidence that schizophrenia is of neurodevelopmental origin^2^,^10^,^11^,^12^. We stress, however, that further research and a more extensive functional understanding of pathways and gene–gene interactions is essential to corroborate this hypothesis.

In our study, we do not observe a relationship between common and rare susceptibility alleles. While the absence of a significant overlap between rare variant genes and GWAS genes (empirical *P*=0.07) may be due to a lack of power, the absence of a correlation between ISUB scores and common variant burden suggests that common and rare disease risk alleles may be independently and additively enriched in cases versus controls. Regarding genes containing previously reported *de novo* mutations in schizophrenia, we also did not observe a difference between overlap in cases and in controls. However, the overlap within each group was highly significant. This may suggest that these previously reported genes are prone to (*de novo*) mutational events in general, but perhaps this is not specific to schizophrenia.

In summary, we observe an increased burden of deleterious coding variants in schizophrenia at a genome-wide level. The genes containing rare coding variants significantly overlap with genes expressed in fetal brain highlighting the potential involvement of neurodevelopment in disease aetiology^11^. Our results are an important step towards a better understanding of the genetic architecture of schizophrenia and the extensive polygenic nature of disease susceptibility, also at the level of rare variants.

## Methods

### Sample description and genotyping

We genotyped 1,042 schizophrenia cases and 961 unaffected controls^1^,^4^,^13^ from a relatively homogeneous Dutch population^14^ using the Illumina HumanExome BeadChip array. In-patients and outpatients were recruited from different psychiatric hospitals and institutions throughout the Netherlands, coordinated via academic hospitals in Amsterdam, Groningen, Maastricht and Utrecht. Detailed medical and psychiatric histories were collected, including the Comprehensive Assessment of Symptoms and History (CASH), an instrument for assessing diagnosis and psychopathology. Only patients with a DSM-IV diagnosis of schizophrenia were included as cases; controls were volunteers, free of any psychiatric history^4^,^5^,^13^. The exome array includes >250,000 putative functional exonic single-nucleotide polymorphisms (SNPs) observed multiple times in whole-genome and exome sequence data from over 12,000 subjects^15^. This array was designed as an intermediate platform between exome sequencing and common SNP arrays for studying relatively rare coding SNPs with MAF ≥0.01 %. For more details on the exome SNP array design see ( http://genome.sph.umich.edu/wiki/Exome_Chip_Design). All samples were genotyped at UCLA Neurosciences Genomics Core with cases and controls randomized on plates. This study was approved by the UCLA Institutional Review Board and all subjects provided informed consent.

### Quality control

We applied the following quality control to our original data set using PLINK(v1.08p)^16^. We excluded samples with ambiguous sex or with imputed sex inconsistent with our database (*n*=20), as well as samples with missing genotyping >5% (*n*=3). On the basis of a set of 13,597 SNPs with a MAF>10%, missing genotype rate of at most 1%, with maximum LD *R*^2^ of 0.2, we excluded samples for too high (>mean+3 s.d.) or too low (<mean−3 s.d.) heterozygosity (*n*=9), as well as samples related up to the level of distant cousins (*n*=37). We excluded one individual based on multidimensional scaling cluster of the two principal components (*n*=1) (Supplementary Fig. 2). On the basis of the resulting set of 1,002 cases and 931 controls, we removed 307 SNPS with a missing genotyping rate >5%, as well as the SNPs located on the X and Y chromosome and mitochondrial SNPs. Finally, we excluded flagged sites as tabulated here ftp://share.sph.umich.edu/exomeChip/IlluminaDesigns/cautiousSites/cautiousSite.sorted.sites

Our subsequent analysis was based on 234,353 remaining autosomal SNPs.

### Variant selection and ISUB score

To quantify the burden of rare coding variants in our sample, we computed an ISUB score using the following method. To begin, variants occurring only in cases (‘case set-unique’ *n*=17,035) and variants occurring only in controls (‘control set-unique’ *n*=13,529) were selected. This set of variants included in ISUB analysis is likely to be a lower bound on the number of actual rare variants in the data set, as it was shown in previous work that the standard variant caller GenCall, developed by Illumina, has a bias towards not being able to call singleton heterozygote variants^17^.

We compared the allele frequency of our set-unique SNVs with the ESP6500 database ( http://evs.gs.washington.edu/EVS/ accessed on 20 October 2014). From 30,564 set-unique variants, a total of 27,544 were included in the database. The mean MAF of the variants is 0.046% based on the population of European ancestry (*n*=4,300, with a median 0.035% s.d. 0.051% min 0.00%, max 1.01%). This implies the average frequency of our variants is around 1:1,000 in the population.

To assign a numeric score to the relative deleteriousness of each variant, we applied a previously developed CONsensus DELeteriousness (CONDEL) scoring algorithm^7^. This consensus scoring metric integrates the output of five existing prediction tools aimed at assessing the impact of non-synonymous SNVs on protein function and holds a value between 0 and 1. These five tools included in the consensus method are SIFT, Polyphen2, MAPP, LogR and Pfam E-value. In addition to assigning a continuous score, the CONDEL algorithm also classifies variants as deleterious or neutral based on a weighted average of the assigned scores.

The array also includes splice site (*n*=12,662) and stop-altering (*n*=7,137) SNVs that we obtained from ftp://share.sph.umich.edu/exomeChip/ProposedContent/codingContent/

Because most of these variants do not cause amino-acid changes, many are not scored by the CONDEL algorithm. To include them in our analysis, we therefore augmented the CONDEL function by assigning a maximal score and a deleterious label all non-scored splice site and stop-altering variants. Finally, for each subject, we computed a burden score as the sum of scores of all observed set-unique non-synonymous SNVs (denoted NS) as well as only the set-unique non-synonymous SNVs predicted to be deleterious (denoted DEL).

### Statistical analysis

The *P* values are estimated empirically by permuting phenotypes within subgroups of cases and controls 10,000 times. For each repetition, we determined the set of unique variants and the individual scores based on the current case-control assignments and computed the Wilcoxon test statistic. This method both corrects for the differences in sample size in cases versus controls and is conservative with respect to potential outliers driving the signal.

Correction for multiple testing was performed empirically using the family-wise ‘minP’ method also employed in Purcell *et al.*^6^. For each permuted version of the data we computed the minimal empirical significance of each data set, and compared the empirical *P* value of a given test to the distribution of minimal *P* values across all tests in each family. This procedure was adopted to preserve family-wise error rates without overcorrection.

The families of tests considered in our analysis are the following: Primary burden analysis (Table 1: One test for all, deleterious, and Benign variants—3 tests), Gene burden analysis (Table 2: One test for each gene set—4 tests), GWAS and *de novo* gene list overlap (Supplementary Table 2—2 tests).

We also present a number of exploratory/descriptive results for which we only report uncorrected *P* values. These include Burden analysis comparing average number of SNVs versus score per SNV (Supplementary Table 1), additional observations presented in the Supplementary Material only.

### Replication data set

A total of 5,585 European ancestry schizophrenia cases and 8,103 controls were used to replicate our main finding. The cases were taken from two collections, Cardiff COGS and CLOZUK, both of which contributed to the recent GWAS study from the Psychiatric Genomics Consortium^1^. Two groups of UK controls were used in this study; UK Blood Service donors (4,455 samples) and the 1958 British Birth Cohorts (4,615 samples)^18^,^19^,^20^. From these subjects we obtained individual minor allele counts of deleterious rare variants identified through ISUB analysis on our data.

### DAVID analysis

We defined a set of ‘differentially hit’ genes as those genes with deleterious variants observed in more than two cases and with an increased burden in cases versus controls using Fisher exact *P* value threshold of 0.5. For controls, the set is defined analogously. In particular, because of gene limits, our analysis excludes those genes affected by a deleterious variant in exactly one or two cases, versus zero controls and vice versa. We have analysed these genes separately (Supplementary Methods).

Standard DAVID ( http://david.abcc.ncifcrf.gov/ version 6.7, accessed January 2015) settings were used for functional annotation of gene sets included in our analysis. Databases included to annotate gene list for tissue expression are GNF_U133A_QUARTILE, PIR_TISSUE_SPECIFICITY and UP_TISSUE and for pathways are BBID, BIOCARTA, EC_NUMBER, KEGG_PATHWAY, PANTHER_PATHWAY, REACTOME_PATHWAY. The full results can be found in Supplementary Table 2.

### Gene list overlap

We tested for the overlap between genes contained in the GWAS associated intervals Schizophrenia Working Group of the Psychiatric Genomics Consortium^1^ (ED Supplementary Table 3). *P* value for the difference in overlap between cases and controls was computed empirically by permutation (see also Supplementary Table 2). We adopted the same analysis to test for genes containing previously reported *de novo* mutations (Supplementary Tables 1 and 2 from Fromer *et al.*^2^).

### Quantifying the contribution of rare variants to schizophrenia

To determine the relative effect size of ISUB variants, we fit the following logistic regression model on a subset of 385 controls and 708 cases included in the GWAS analysis^1^




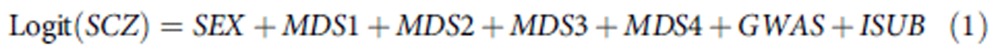




where *MDSi* indicated the *i*th multidimensional scaling component, based on 13,597 independent common SNPS (see Quality Control).

*GWAS* is the polygenic risk score with a *P*-value cutoff of 0.05 with our samples removed^13^.

*ISUB* is the deleterious ISUB score, corrected for sample size by multiplying the scores of cases by 
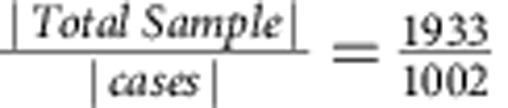
 and analogously for controls, and normalized by inverse normal transformation.

Variation explained by ISUB as well as polygenic risk scores (*GWAS*) are measured by the reduction in *R*^2^ comparing the full logistic regression model versus a reduced model with that term removed, as proposed by Nagelkerke^21^.

## Additional information

**How to cite this article:** Loohuis, L.M.O. *et al.* Genome-wide burden of deleterious coding variants increased in schizophrenia. *Nat. Commun.* 6:7501 doi: 10.1038/ncomms8501 (2015).

## Supplementary Material

Supplementary Figures, Tables, Methods and References Supplementary Figures 1-2, Supplementary Tables 1-3, Supplementary Methods and Supplementary References

Supplementary Data 1 Genes including set-unique deleterious variants. List of genes including at least one set-unique deleterious variant in cases or controls; the number of deleterious set-unique SNVs observed in them; the number of individuals carrying at least one such variant; and the fisher exact P-value of case/control difference (cases n=1,002, controls n=931).

Supplementary Data 2 DAVID analysis of genes including set-unique variants. Table showing DAVID results of significantly enriched tissues and pathways (ordered by Benjamini corrected P-value) for differentially hit genes. Values included are database category, tissue, number of genes from the input set included in the category, uncorrected P-values determined by DAVID based on hyper-geometric test, and Benjamini corrected P-values.

## Figures and Tables

**Table 1:** Individual set-unique burden (ISUB) analysis.

**Variant type**	**Disease Status**	**Number of SNVs**	**Individual burden score (mean/median/s.d.)**	***P*** **value**
NS	Case	16,262	8.73/ 7.26/ 9.65	**0.033**
	Control	12,964	7.27/ 6.56/ 5.62	
DEL	Case	6,424	7.54/ 6.29/ 8.18	**0.018**
	Control	5,130	6.23/ 5.58/ 4.78	
NS—DEL	Case	9,838	1.18/ 0.94/ 1.62	0.492
	Control	7,834	1.04/ 0.85/ 1.07	

The number of set-unique SNVs and mean individual burden score for all non-synonymous variants (NS) as well as the two complementary subsets of only deleterious (DEL) variants as well as non-synonymous variants not predicted to be deleterious (NS-DEL). Empirical *P*-values are estimated by permutation (10,000 permutations) of the phenotypes (cases *n*=1,002, controls *n*=931) based on Wilcoxon Rank Sum Test statistics. See also Supplementary Fig. 1 and Supplementary Table 1. Values in bold withstands correction for multiple testing (Supplementary Methods).

**Table 2:** Set-unique burden of different variant types.

**Variant Type**	**Disease status**	**Total genes**	***P*** **value**
Deleterious genes	Case	4,533	**0.008**
	Control	3,795	
Splice site	Case	380	**0.016**
	Control	276	
Stop-altering	Case	350	**0.004**
	Control	253	
Double hits	Case	179	0.047
	Control	103	

The polygenic burden of deleterious hits, splice site and stop-altering variants and double hits arising from set-unique SNVs (cases *n*=1,002, controls *n*=931). The category of double hits includes those genes for which an individual has two or more SNVs (of which at least one is predicted to be deleterious) within the borders of the gene. Each row contains the number of genes having at least one variant. Empirical *P* values for the difference in gene count are estimated empirically by permutation of phenotypes. Values in bold withstand correction for multiple testing (Supplementary Methods).
