# Supplementary material for: Genome-wide burden of deleterious coding variants increased in schizophrenia
Source: Nat Commun. 2015 Jul 9;6:7501. doi: 10.1038/ncomms8501 (PMC4499856; doi:10.1038/ncomms8501)
Supplement: Supplementary Figures, Tables, Methods and References — Supplementary Figures 1-2, Supplementary Tables 1-3, Supplementary Methods and Supplementary References [file ncomms8501-s1.pdf]

Supplementary Figures

Supplementary Figure 1a

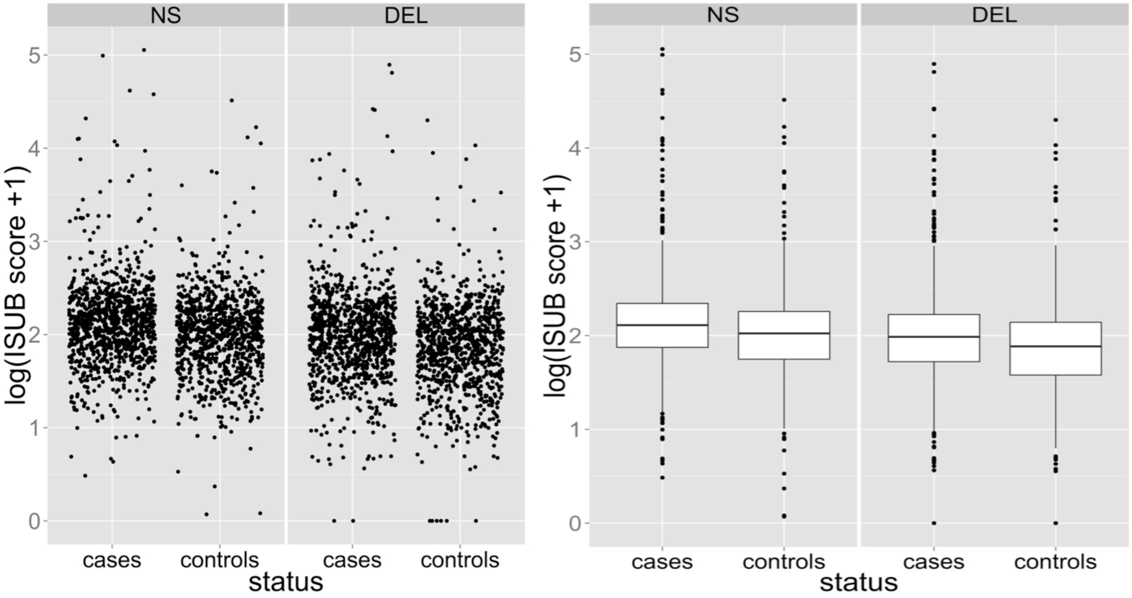

Supplementary Figure 1b

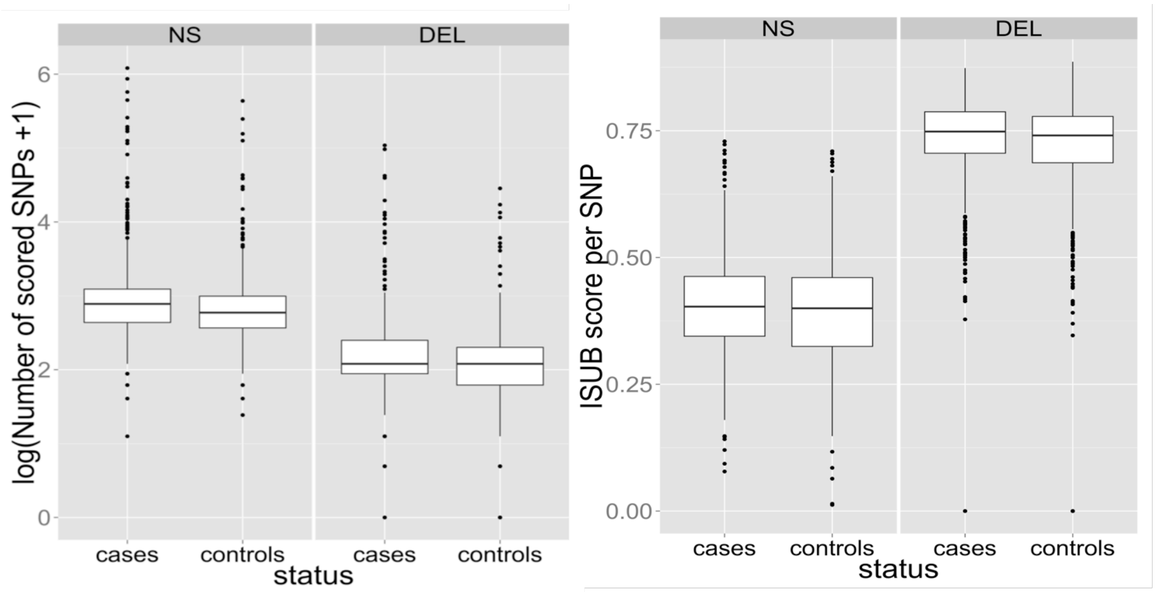

Supplementary Figure 1c

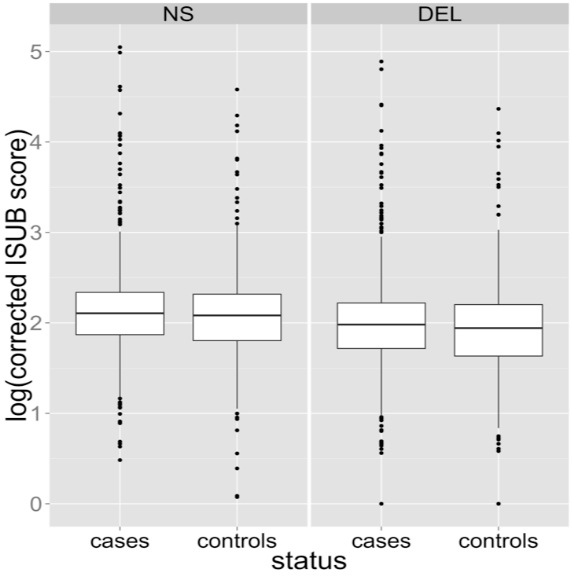

Supplementary Figure 1 | Individual set-unique burden
Scatterplot and boxplot of ISUB scores (n=1002 cases and n=931 controls, logarithmic scale, upper and lower hinges correspond to the 25th and 75th percentiles, and whiskers to 95% intervals) (**a**) Boxplot of total number of scored SNVs and individual score per SNV for both NS (the set of all non-synonymous set-unique variants, left) and DEL (the set of deleterious set-unique variants, right) categories (**b**). We also show a boxplot (logarithmic scale) of the individual scores corrected for sample size (**c**). That is, for each case the score shown is the actual score multiplied by $\frac{|\boldsymbol{Total\ Sample}|}{\boldsymbol{2|cases|}} = \frac{\mathbf{1933}}{\mathbf{2004}}$ and analogously for controls. Note that this is purely for illustrational purposes as our statistical analysis controls for differences in sample size.

Supplementary Figure 2a

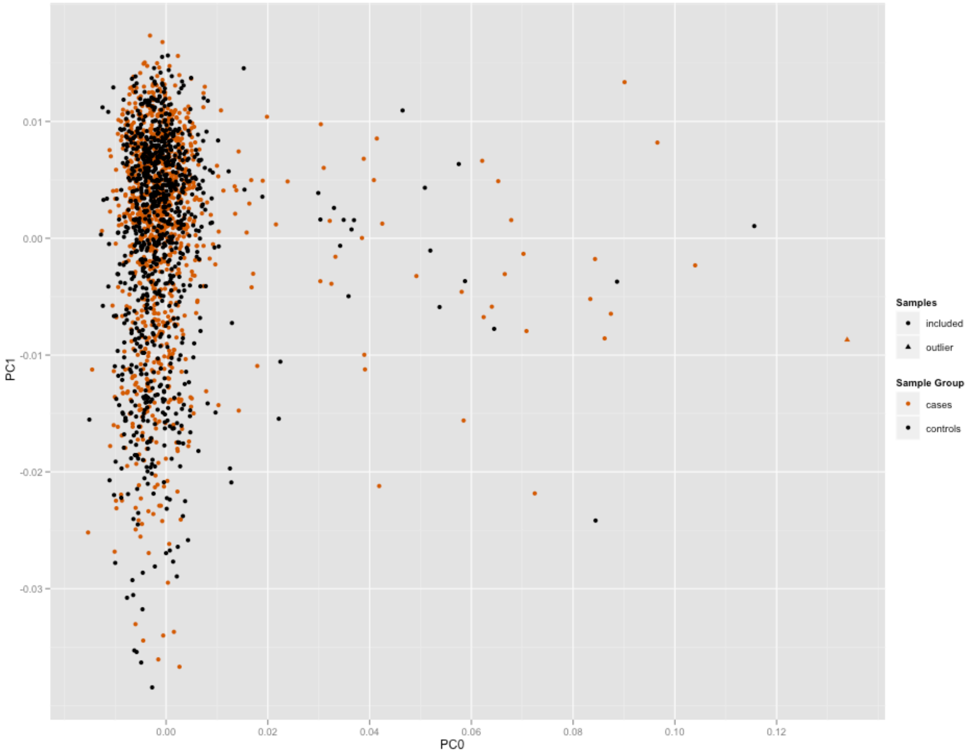

Supplementary Figure 2b

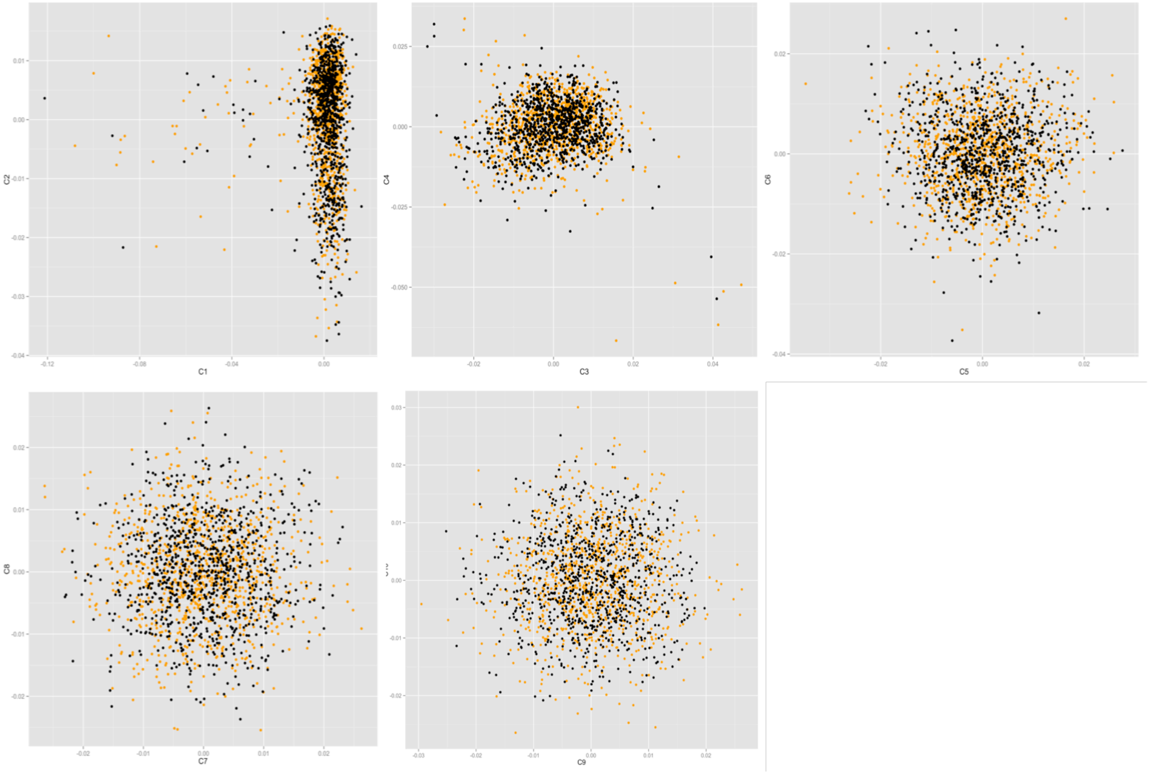

Supplementary Figure 2c

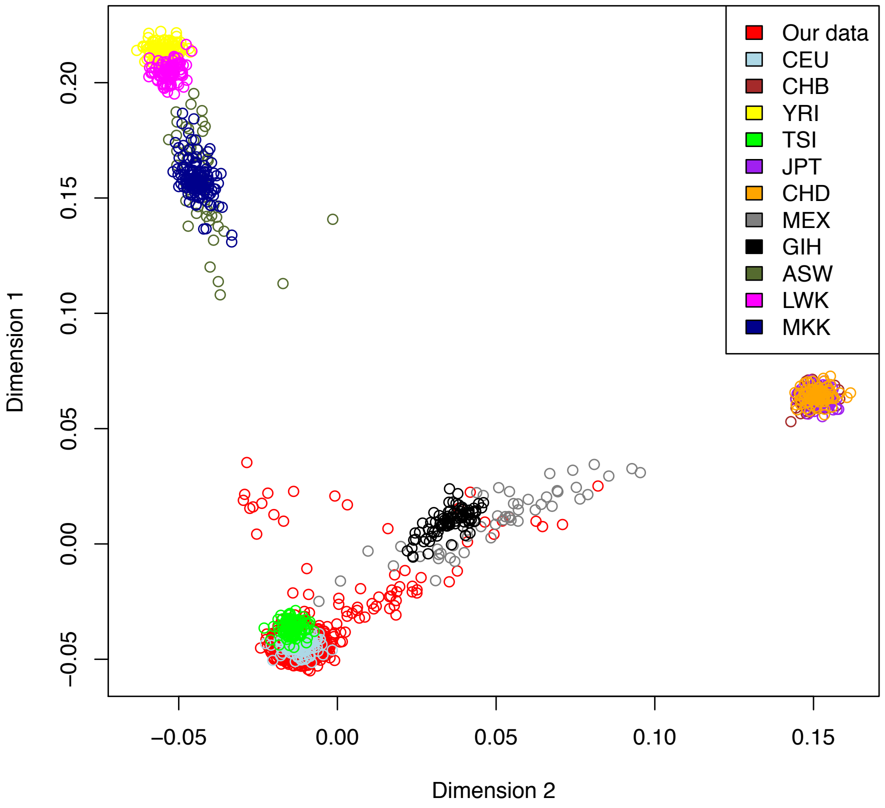

Supplementary Figure 2 | Multi Dimensional Scaling (MDS) plot of the 1934 samples

(a) MDS plot of 1,934 samples: 1,003 cases (orange) and 931 controls (black) showing the first two principal components. The rightmost case (triangle) was considered an outlier and excluded from our analysis. (**b**) MDS plots of the first ten principal components of the remaining 1,933 samples based on common independent (MAF>1%, LD R^2 < 0.2, n=13,400) SNPs included in our analyses. (**c**) MDS plot of the first two principal components of our samples combined with HapMap3 samples based on common independent SNPs overlapping with the HapMap (n=8,174 SNPs).

Supplementary Tables

Supplementary Table 1 | Extended ISUB analysis

Total number of set-unique SNVs, individual burden score as well as total number of scored SNVs for non-synonymous deleterious variants (DEL). Empirical p-values are estimated by permutation based on Wilcoxon Rank Sum test statistics. (See also Extended Data figure 1). Values in bold indicate significant findings.

Variant Type	Disease Status	SNVs	Number of Scored SNVs (mean /median/ sd)	Individual Burden Score (mean/median/sd)	P-value ISUB score	P-value Individual score per SNVs	P-value Individual number of scored SNVs	P-value Total number of SNVs - group level
**DEL**	case<br>control	6,424<br>5,130	8.89/7/9.72<br>7.34/7/5.70	7.54/ 6.29/ 8.18<br>6.23/ 5.58/ 4.78	**0.018**	0.079	**0.015**	**0.015**

Supplementary Table 2 | Overlap with GWAS and *de novo* genes

Overlap between genes containing deleterious mutations and genes contained in the GWAS associated intervals (Schizophrenia Working Group of the Psychiatric Genomics Consortium[1] Extended Data Table 3), as well as genes containing reported *de novo* variants in schizophrenia (Supplementary Tables 1 and 2 from Fromer, et al.[2]). All genes with at least one set-unique deleterious variant are included. Empirical P-values of the overlap difference between cases and controls are estimated by permutation. Empirical p-value in bold withstands correction for multiple testing.

Variant Type	Genes Included	Disease Status	Genes	Overlap	P-value difference (empirical)	P-value overlap (hyper-geometric)
**GWAS (316)**	**All DEL**	**case**	4,533	83	0.070	0.29
		**control**	3,795	62		0.72
***De novo* (832)**	**All DEL**	**case**	4,533	315	0.070	**<2.2e-16**
		**control**	3,795	270		**4.44e-16**

Supplementary Table 3 | DAVID analysis of permuted data sets

	cases #enriched datasets	controls #enriched datasets	P-value Fisher
**Tissue fetalbrain**	31	2	**1.14E-08**
**Pathway ECM**	59	0	**< 2.2e-16**

Table summarizing DAVID results from a permutation analysis using an equal number of cases and controls (931 for both) on 100 datasets. For each such dataset, the number of times fetalbrain and the pathway ECM receptor interaction were significantly enriched after Benjamini correction are reported. P-values are computed using Fisher exact test.

Supplementary Methods

Assigning individual burden scores

To assign a score to the relative deleteriousness of single nucleotide variants, we incorporate the CONsensus DELeteriousness score of non-synonymous SNVs (accessed November 2013)[3]. This scoring metric integrates the output of five existing computational tools aimed at assessing the impact of non-synonymous SNVs on protein function and holds a value between 0 and 1. These five tools included in the consensus method are: SIFT, Polyphen2, MAPP, LogR and Pfam E-value. In addition to assigning a continuous score, the CONDEL algorithm also classifies mutations as deleterious or neutral based on a weighted average of the assigned scores. In addition to non-synonymous SNVs, the exome array also includes a number of splice site (n = 12,662) and stop-altering (n = 7,137) SNPs. While the CONDEL algorithm scores non-synonymous variants located on the exon, the algorithm does not score stop and many of the splice site variants (a total of 6,679/12,662 are scored by CONDEL). Because we wish to include these variants in our analysis we have augmented the CONDEL function with these variants, by assigning a score of 1 to each non-scored stop-altering or splice site variant. We thus work under the assumption that these variants are likely disruptive, and classify them as deleterious.

On the basis of each scored SNV we compute an ISUB score for each individual by summing the maximum scores of each SNV the individual carries. Note that the score of each variant is added to the individual score only once regardless of whether the individual is homozygous or heterozygous for that variant.
Formally, for each individual *i* the score $\mathrm{C}(i)$ is defined as follows:

$$\mathrm{C}(i) = \sum_{s\epsilon \mathrm{U}(i)} \max\,[\mathrm{c}(s)], \quad (1)$$

where i is the individual, $\mathrm{U}(i)$ is the set of set-unique variants that i carries, s is a SNV and c is the (overloaded and augmented) CONDEL function assigning a score (or set of scores) to every non-synonymous SNV. In addition, we computed a second individual score including only *deleterious* mutations identified by the method.
Two points should be noted at this point: First, some SNV receive multiple scores. For example, if the functional impact of a mutation is affected by alternative splicing. In this case we include only the maximum CONDEL score for each variant (max). Second, some SNVs receive no score. This may be due to the fact that either the SNP is located is non-coding region, or the particular variant may be a synonymous variant. Out of our original 17,035 case set-unique SNVs, 16,262 were scored including 390 splice site variants not scored by CONDEL as well as 360 stop altering variants. For controls, these numbers are 13,529 total variants, 12,964 scored variants including 280 splice site, and 262 stop altering variants.

Statistical analysis

To evaluate whether schizophrenia cases carry an increased burden of rare variants, we computed a Wilcoxon Rank Sum Test of the ISUB scores and determined P-values empirically by permuting phenotypes within subgroups of cases and controls 10,000 times. For each repetition, we determined the set of unique variants and the individual scores based on the current case-control assignments and computed the Wilcoxon test statistic. By computing P-values empirically we avoid controlling for at least two issues arising from the fact that we have a larger group of cases compared to controls. First, one expects the set of unique variant in cases to be greater than controls by virtue of sample size alone, thus leading to a larger set of scored variants per person and thus a higher individual score. Second, because rare variants are hypothesized to be more deleterious on average than common ones, it may be that because of the larger case set, the unique control samples are more unique and therefore, on average, more deleterious than set-unique case SNVs. In addition, we use the Wilcoxon rank sum test rather than a Student's T-test or a similar mean-based test performed on normalized data, to avoid picking up a signal that is carried by relatively few individuals. Correction for multiple testing was performed empirically using the family-wise "minP" method (Methods).

Hardy Weinberg equilibrium

For two reasons, we do not exclude SNVs that are out of Hardy Weinberg equilibrium (HWE) in our analysis: First, since traditionally only SNVs are excluded that are not in equilibrium in *controls*, we believe that excluding these variants introduces a bias towards more unique variants in cases versus controls. Second, due to the low frequency of rare variants, any homozygous mutation is out of HWE ($P < 0.0005$).

However, the variants out of HWE do not seem to significantly contribute to our results. First, we observe very similar results when we remove the variants out of HWE both in cases and in controls (i.e, the empirical P-value for increased burden score for the set DEL is now 0.021 compared to 0.018 in our original result). Second, if we observe a homozygous variant we only add the score of this variant once to the total individual score. The total number of set-unique variants out of HWe ($P < 0.001$) is 699 in cases, versus 535 in controls (a proportional number given the total number of set-unique variants, $P = 0.52$ fisher exact test).

Quantifying the contribution of set-unique variants to schizophrenia

To determine the relative effect size of ISUB variants, we fit the following logistic regression model on a subset of 385 controls and 707 cases included in the GWAS analysis[1]:

Logit(*SCZ*) = *SEX* + *MDS1* + *MDS2* + *MDS3* + *MDS4* + *GWAS* + *ISUB* (1)

Where:

MDSi are the MDS components

GWAS is the polygenic risk score with a P-value cutoff of 0.05 with our samples removed[4].

ISUB is the deleterious ISUB score, corrected for sample size by multiplying the scores of cases by $\frac{|Total\ Sample|}{|cases|} = \frac{1933}{1002}$ and analogously for controls, and normalized by inverse normal transformation.

In this subset of samples, both *ISUB* and *GWAS* (and none of the MDS components) are significant predictors of disease risk after inclusion of all other terms (at a threshold of <0.05 Wald test). The GWAS predictor explained an order of magnitude more variation as measured by the reduction in R^2 comparing the full logistic regression model versus a reduced model with that term removed[5]: *GWAS* $R^2 = 0.107$ *ISUB* $R^2 = 0.006$, in line with observations from Purcell, et al.[6] This result also indicates that effects of population stratification do not drive the increased burden we observe. Analogous results are obtained if we fit the model

$$\text{Logit}(SCZ) = SEX + MDS1 + ... + MDS10 + ISUB$$

using our whole dataset, including sample for which we do not have polygenic risk scores (1,002 cases and 931 controls).

Excluding genes previously associated with schizophrenia

To assess the polygenic width of the observed signal, we obtained a list of genes prominently enriched for rare disruptive mutations in previous work[6] (from SI Table 2, n = 1,796). We performed our original analysis while excluding this list of genes and continued to observe increased burden from deleterious variants (DEL, empirical P = 0.006) as well as all set-unique non-synonymous variants (NS, empirical P = 0.009).

Polygenic nature of the increased burden

P-values of the difference between the total number of genes affected by deleterious variants, double hits, splice site and stop altering variants are determined empirically by permutation of phenotypes. Due to the low frequency of set-unique double hits, stop-altering and splice site variants, the larger number of double hits in schizophrenia is observed at the group level, in contrast to the polygenic burden at the level of the individual.

Splice site variants located on the exon can potentially result amino-acid changes and may be scored by the CONDEL algorithm. In addition to genes affected by uniquely splice site variants (n = 5,983) not scored by CONDEL, we also studied this second type of splice/non-synonymous variants (n = 6,679). If we include both variant types in our analysis, a total of 704 genes harbor splice site mutations in cases, compared to 523 in controls (empirical P = 0.007). The signal appears to be driven both by non-amino-acid

changing variants (353 genes in cases, versus 261 in controls, empirical *P* =0.015) as well as splice/non-synonymous variants (380 genes in cases, versus 276 in controls, empirical *P* = 0.016).

The stop-altering variants include both stop-gain (n = 6,714) and stop-loss (n = 425) mutations (two variants are tri-allelic resulting in either a stop-gain or stop-loss variant).

To study genes containing potential compound heterozygous missense SNVs we included only those genes containing two or more SNV of which at least one deleterious in the same individual within the borders of the gene. In the absence of genotyping data from family members and because of the nature of the exome SNP array it is not possible to discriminate between actual compound heterozygous mutations and two variants on the same strand. However, we expect that a large proportion of the SNVs actually represent compound heterozygous mutations.

While splice site and stop-altering variants show a strong signal, this signal does not appear to be driving the overall increase in burden. For example, ISUB analysis of purely non-synonymous deleterious variants scored by CONDEL alone show similar results (DEL, empirical *P* = 0.036). In addition, the increased burden from these variants appears to be larger than the overall increase in burden, and therefore not likely to be caused by it (empirical *P* = 0.033 splice site, empirical *P* = 0.035 stop altering, empirical *P* = 0.001 double hits; one-sided Fisher exact test). The genes affected by deleterious ISUB variants are not different in cases versus controls (mean = 81Kb in controls versus 76Kb in cases, *P* = 0.12 Wilcoxon Ranksum test)

Gene selection for DAVID analysis

We defined a set of "differentially hit" genes as those genes with more than two deleterious set-unique SNVs observed in cases and with an increased burden in cases versus controls using Fisher exact P-value threshold of 0.5. For controls, the set is defined analogously. The set of differentially hit genes is defined analogously for controls.

This category is not only useful to reduce our set of included genes to a number acceptable for DAVID analysis (< 3,000), it also excludes a set of genes that are observed to be highly polymorphic even at the level of rare variants in our dataset. These genes include *NEB* (12 controls and 9 cases have at least one deleterious SNV in this gene) and *MUC16* (6 controls, 12 cases). Whether these genes are actually polymorphic (and perhaps dispensable), or these observations are due to artifacts of data-collection, we believe that the signal coming from the selected gene sets includes less noise. For a full list of genes, the number of deleterious set-unique SNVs observed in them, and the number of individuals carrying at least one of such variants, see Supplementary Data 1.

The total set of selected genes for cases includes 698 genes (of which 687 have a valid DAVID ID) and 513 for controls (510 with a DAVID ID).

In particular, because of gene limits, our analysis excludes those genes affected by a deleterious variant in exactly one or two cases, versus zero controls and vice versa. We have analyzed these genes (N = 2,350 in cases, and N = 1,848 in controls) separately.

Functional annotation analysis using DAVID.

Standard DAVID (http://david.abcc.ncifcrf.gov/ version 6.7, accessed January 2015) settings were used for functional annotation of gene sets included in our analysis. Databases included to annotate gene list for tissue expression are GNF_U133A_QUARTILE, PIR_TISSUE_SPECIFICITY and UP_TISSUE and for pathways are BBID, BIOCARTA, EC_NUMBER, KEGG_PATHWAY, PANTHER_PATHWAY, REACTOME_PATHWAY. The full results can be found in Supplementary Data 2.

Tissue expression analysis of the gene sets shows significant enrichment for fetal brain in cases (i.e. 117 of 698 genes are expressed in fetal brain; uncorrected *P* = 0.001, Benjamini corrected *P* = 0.034, hypergeometric overlap test). This enrichment highlights existing evidence that schizophrenia is of neurodevelopmental origin. To control for potential confounders such as gene length, we performed the same analysis for controls, and while certain enrichments were observed (Supplementary Data 2), neither of the above regions were significantly enriched in controls. In addition, DAVID pathway analysis demonstrates the extracellular matrix (ECM) receptor *interaction* as the only significantly enriched pathway in cases (*P* = 5.89E-05, Benjamini corrected *P* = 0.008, hypergeometric overlap test), whereas no pathway enrichment was observed in controls. While this finding is tentative, it may provide support for evidence of involvement of ECM signaling in brain function[9].

Other than gene length – which is controlled for by comparing results across cases and controls, one may argue that the selection of genes introduces another type of bias: An increased number selected genes in cases is expected partly due to differences in sample size. Since the genes in our analysis are sparsely hit by deleterious ISUB variants (i.e. most genes are hit exactly by one variant in a single case or control) we are unable to select genes using relative enrichment P-values. Moreover, since DAVID analysis highlights *relative* enrichment given the number of input genes, we do not expect the larger number of genes in cases to affect the outcome of our results. However, to investigate this issue quantitatively, we performed a permutation analysis using an equal number of cases and controls (931 for both) on 100 datasets. For each such dataset, we computed the set-unique variants as well as the differentially hit genes and found fetalbrain as well as the pathway ECM receptor interaction to be consistently enriched in cases compared to controls (Supplementary Table 3).
Our analysis of the genes affected only once or twice in cases or controls but not both yielded only Liver as a significantly enriched for tissue expression (*P* = 2.70E-04, Benjamini corrected *P* = 0.021, hypergeometric overlap test),

Gene list overlap

We tested for the overlap between genes contained in the GWAS associated intervals Schizophrenia Working Group of the Psychiatric Genomics Consortium[1] (Supplementary Table 3) and the genes containing deleterious mutations using the following method. A total of 18,264 genes were included on the array after QC (these are the set of genes included in our analysis). From the set of GWAS genes, a total of 316 is included in this list, of which 83 overlap the 4,533 genes in cases. In controls a total of 62/3,795 overlap the GWAS genes. We determined a P-value for the difference between cases and controls (>20 genes) empirically by permutation (empirical P = 0.070, see also Supplementary Table 2).

We adopted the same analysis to test for genes containing previously reported *de novo* mutations (SI Tables 1 and 2 from Fromer, et al.[2]). From this set of *de novo* genes, a total of 832 genes overlapped with genes contained in the SNP array. A total of 315/4,533 and 270/3,795 overlapped this list in cases and controls respectively, while the difference between cases and controls is not significant (empirical P = 0.07), overlap measured by hypergeometric overlap test indicates significant overlap both for cases and controls (P = < 2.2E-16 cases P = 4.44E-16 controls, hypergeometric overlap test).

We have further examined the nature of this enrichment of deleterious coding variants in both cases and controls of genes with observed de novo mutations. First, we addressed the potential bias of gene length. We took the list of genes represented on the exomeSNP array and divided these genes whose Refseq length is shorter than the median of all genes included on the array and those with length greater than the median. We performed the same enrichment analysis for the genes smaller than the median length and observe a similar enrichment of deleterious variants in genes on the de novo list for both cases (P = 2.83E-6, hypergeometric overlap test) and controls (P = 0.004, hypergeometric overlap test). If we examine the long genes only (longer than the median), the results are significant as well (P = 5.88E-11 cases, P = 6.02E-13 controls, hypergeometric overlap test).
Second, we identified the genes with deleterious rare variants in cases only (n = 2,880), in controls only (n = 2,142), and those that are shared between cases and controls (n=1,653). For each of the sets we tested for the overlap with the genes from the de novo list. We observe that the overlap is primarily driven by genes with rare variants in both cases and controls (P= 0.01 for cases only, P= 0.02 for controls only, P < 2.2E-16 both, hypergeometric overlap test).

Thus, these results suggest that while there is no difference between cases and controls, the enrichment of deleterious coding variants in genes from the de novo list holds true both from short as well as long genes and is primarily driven by genes with deleterious variants observed in both cases and controls.

We also observe an overlap of three (*LAMA2, DPYD, VPS39*) out of four genes (*LAMA2, DPYD, TRRAP, VPS39*) affected by recurrent de novo events as presented in Xu, et al.[7] compared to only TRRAP in controls.

Relation between ISUB score and Sex

Given recent evidence of an increased mutational load in females in neurodevelopmental disorders[8], we hypothesized a higher rare variant burden in females than males. However, we did not observe a difference in ISUB score of deleterious variants between male (n = 741) and female (n = 261) cases (DEL, $P = 0.98$, Wilcoxon Rank Sum Test).

Relation between ISUB score and Polygenic Risk Score

To determine a correlation between polygenic risk score and ISUB score for deleterious SNVs, we compared both scores for 707 of our cases included in the most recent genome wide association study[11]. The polygenic risk score was computed using a P-value cut-off of $P < 0.05$, with our samples removed[4]. We observed no correlation between the two scores (Kendall's rank correlation, tau = 0.001, $P = 0.95$), suggesting that either the two mechanisms work independently, or there is not enough power in our sample set to establish any correlation. This result remains if we use different P-value cutoffs to determine the polygenic risk score.

Supplementary References

1. Schizophrenia Working Group of the Psychiatric Genomics Consortium. Biological insights from 108 schizophrenia-associated genetic loci. *Nature* **511**, 421--427 (2014).
2. Fromer, M. *et al.* De novo mutations in schizophrenia implicate synaptic networks. *Nature* **506**, 179-84 (2014).
3. Gonzalez-Perez, A. & Lopez-Bigas, N. Improving the assessment of the outcome of nonsynonymous SNVs with a consensus deleteriousness score, Condel. *Am J Hum Genet* **88**, 440-9 (2011).
4. Ripke, S. *et al.* Genome-wide association analysis identifies 13 new risk loci for schizophrenia. *Nat Genet* **45**, 1150-9 (2013).
5. Nagelkerke, N.J. *Maximum likelihood estimation of functional relationships*, (Springer, 1992).
6. Purcell, S.M. *et al.* A polygenic burden of rare disruptive mutations in schizophrenia. *Nature* **506**, 185-90 (2014).
7. Xu, B. *et al.* De novo gene mutations highlight patterns of genetic and neural complexity in schizophrenia. *Nat Genet* **44**, 1365-9 (2012).
8. Jacquemont, S. *et al.* A higher mutational burden in females supports a “female protective model” in neurodevelopmental disorders. *The American Journal of Human Genetics* **94**, 415-425 (2014).
